# Supplementary material for: Understanding the implementation of continuity-enhancing innovations as steps towards midwife-led continuity of care: A qualitative study using Normalization Process Theory
Source: PLoS One. 2026 Apr 21;21(4):e0347791. doi: 10.1371/journal.pone.0347791 (PMC13098977; doi:10.1371/journal.pone.0347791)
Supplement: S1 File — (DOCX) [file pone.0347791.s001.docx]

Supplement material 1 – Overview of the COMIC project

The current study is part of the larger Continuity Of MIdwifery Care (COMIC) project, which aims to gradually implement Midwife-Led Continuity of Care (MLCC) within the Dutch maternity care system. For a sustainable implementation, in this project different methodological approaches are combined to better understand what supports or hinders MLCC in practice, for whom, and under what circumstances.

The COMIC project is structured into three main phases:

**Realist Review**In the first phase, a realist review was conducted to synthesise international literature from high-income countries on what works, for whom, and under what circumstances, when implementing MLCC. This review identified key mechanisms and contextual factors influencing implementation, with particular attention to power dynamics and professional boundaries.(1)

**Qualitative Study Using Normalization Process Theory (NPT)**The current article presents findings from the second phase of the project. Drawing on interviews with a wide range of stakeholders across different maternity care networks in the Netherlands, we used Normalization Process Theory to analyse how elements of MLCC were implemented in daily practice. This study focused on regions where some degree of MLCC had already been implemented and highlighted how trust and financial feasibility influenced the implementation process.

**Realist Evaluation in Practice-Based Settings**In the third and ongoing phase, we are conducting a realist evaluation in selected regions where new elements of MLCC are actively being implemented. This phase applies lessons learned from both the realist review and the NPT-informed qualitative study to support the implementation efforts. Through an iterative approach involving local stakeholders, we aim to refine and test context-mechanism-outcome configurations and identify actionable strategies for scaling up MLCC in different contexts.

For more information about the COMIC project, please contact the corresponding author.

1. Simmelink R, Neppelenbroek E, Pouwels A, van der Lee N, Pajkrt E, Ziesemer KA, et al. Understanding how midwife-led continuity of care can be implemented and under what circumstances: a realist review. BMJ Open. 2025;15(2):e091968.
