# Supplementary material for: Understanding the implementation of continuity-enhancing innovations as steps towards midwife-led continuity of care: A qualitative study using Normalization Process Theory
Source: PLoS One. 2026 Apr 21;21(4):e0347791. doi: 10.1371/journal.pone.0347791 (PMC13098977; doi:10.1371/journal.pone.0347791)
Supplement: S2 File — (DOCX) [file pone.0347791.s002.docx]

Supplement material 2 - Interview guide maternity care stakeholders

Research question:

- What are the factors that promote and hinder the implementation of full continuous midwife-led care during pregnancy, childbirth and the postpartum period within Maternity Care Networks (VSVs) or Integrated Maternity Care Organizations (IGOs)?

Introduction:

- Purpose of the interview
- Anonymity and confidentiality
- Audio recording
- Informed consent

Background data:

- How long have you been working as an obstetrician / (hospital-based) midwife / manager?
- What is your current role or position?
- How long have you been working in maternity care?
- How long have you been working within this VSV/IGO?
- Age

| **Topics** | **Sub topics** |
| --- | --- |
| What was the reason for the implementation of..... (healthcare innovation) | - Who came up with the initiative? - Dependence on individual initiatives / initiators - Own role - Involvement of the entire Network - Was it part of Best Practices carousel? |
| Who were involved in the project group / working group, and how did it function? | - Composition - Who established the group - Tasks / division of tasks |
| What did the change process look like? | - What exactly has changed? - How long did the process take? - Maintain motivation - Monitoring outcomes - Mid-term evaluations - Feedback to entire Network - Had/missed external support? - Use of implementation tools |
| What practical adjustments have been made? | - Involvement of other disciplines - External project leader - Development of protocols - Working arrangements - Digital support/information |
| How and in what way was ... (healthcare innovation) communicated to other practices / hospitals? | - Positive / negative influence of practices or hospitals from the region - How was this managed? |
| How and in what way was .... (care innovation) communicated to clients? | - By whom - Way of working - Changes - Potential health benefits |
| To what extent did .... (care innovation) contribute to the realization of continuous midwife-led care? | - Continuity during antenatal, intrapartum, and postnatal periods |
| What factors contributed to the implementation of .... (healthcare innovation)? | - Support from policy makers, managers, regional cooperation initiatives - Shared vision - Degree of interprofessional collaboration - External project leader - Financing |
| What barriers or challenges were encountered? | - Expectations versus reality - Financial constraints - Difference in views/interests within the region - Capacity problems |
| What are the experiences with ...... (healthcare innovation) and to what extent have outcomes and experiences been measured and evaluated? | - Personal experiences - Impact on healthcare outcomes - Impact on care processes and optimal use of medical interventions - Registration of outcomes - Satisfaction among midwives - Client satisfaction - Relationships between healthcare providers within the region |
| How do you envision the future of .... (healthcare innovation)? | - Ideal situation - Role of secondary and tertiary care - Role of integrated care - Role of integrated financing - Role of the Royal Dutch Organization of Midwives (KNOV) - Role of regional and national policymakers - National upscaling |
| Do you have any tips for other regions? |  |
| Do you have any other comments? |  |
