# Supplementary figures and images for: Understanding the implementation of continuity-enhancing innovations as steps towards midwife-led continuity of care: A qualitative study using Normalization Process Theory
Source: PLoS One. 2026 Apr 21;21(4):e0347791. doi: 10.1371/journal.pone.0347791 (PMC13098977; doi:10.1371/journal.pone.0347791)

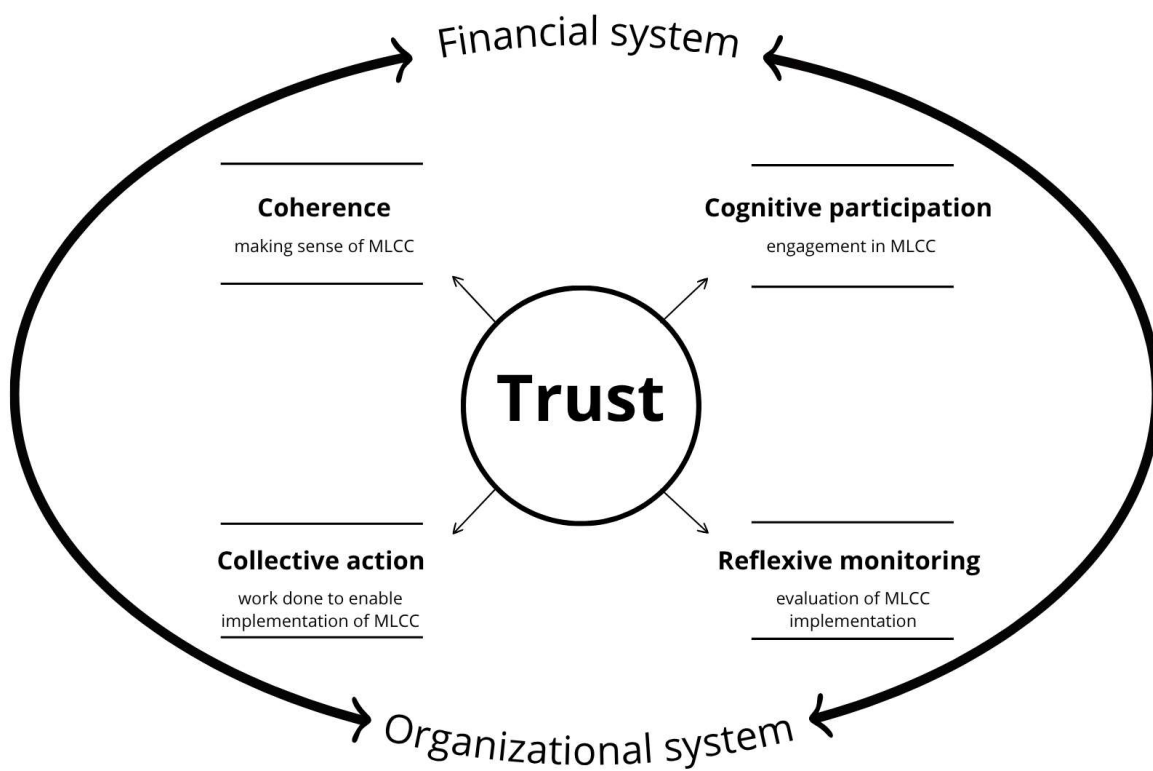

*Figure 1 - Financial system as a requirement and trust as a central theme.*

Supplement: S1 Fig — (PDF) [file pone.0347791.s004.pdf]
