# Supplementary material for: Understanding the implementation of continuity-enhancing innovations as steps towards midwife-led continuity of care: A qualitative study using Normalization Process Theory
Source: PLoS One. 2026 Apr 21;21(4):e0347791. doi: 10.1371/journal.pone.0347791 (PMC13098977; doi:10.1371/journal.pone.0347791)
Supplement: Attachment — Submitted filename: Peer-review comments Nov-25.docx [file pone.0347791.s005.docx]

# **Peer-review of ”Understanding the implementation of midwife-led continuity of care using the Normalization Process Theory”**

- **Most important information**
  - **Summary of the research and your overall impression**

Thank you for the opportunity to review this paper using the Normalization Process Theory to understand the implementation of midwife-led continuity of care. The paper is well written, and using the NPT theory as a conceptual framework to understand how MLCC can become embedded and sustained in everyday work is a good approach. The results are significant for further implementation of MLCC. However, the authors need to **clearly define** that what you have assessed is not a full implementation of MLCC. While this nuance is mentioned in places- such as in the Supplementary material 1, where you note that ”some degree of MLCC had been implemented” – the introduction and methods sections currently give the impression that a full MLCC model was implemented.

- **Major issues that must be addressed**

The Introduction section gives an overview of the midwifery continuity of care (MLCC) model, but when reading the results I understand that this is more about task shifting or shifting care to community settings, and not really about implementing a MLCC model with a small group of midwives providing continuity 24/7 throughout the continuum of care with on-call for their women. The results are important and well-written but the Introduction needs to align better with the rest of the paper i.e. to focus more on midwifery led care and less on the results of the MLCC model (ref 2-4), as this is not the intervention you have explored. A MLCC model also have a strong midwifery philosophy with a focus on women-centred care and less use of unnecessary interventions, and I think the results do not align with the full concept of the model. I feel that using the word MLCC is not really correct when you only talk about part of this concept, i.e. shift in care towards midwife-led settings, and I wonder what the participants discuss when you describe that they had a strong conceptual support for MLCC.

In line 96-98, the authors describe how care is provided for low-risk women but it needs to be added the degree to which continuity is provided. Does the woman meet the same or a few midwives in this model?

In line 133-134, the authors need to clearly define what you mean with ”full continuity of care”.

It is not clear how healthcare is financed in the Netherlands, which makes it a bit confusing to read about financial aspects in the results. There is a short description of the financial system in the discussion (*In systems based on fee-for-service payment models, like in the Netherlands,…)* but this need to be described in the Introduction for the reader to understand the problems described in the Results.

It is also not clear what role health insurance companies have. Please describe this in the background/context.

In line 95, the authors also mentioned what professions are working in the Dutch maternity care system, but it would be helpful if you could describe what role the obstetric nurse has in relation to the midwife.

- **Minor issues to address**

Research question: Since the authors have only used one research question, I recommend changing it to an aim instead.

Methods

Design: Add a reference to the SRQR checklist.

Setting, participants and data collection: Line 123, …*that implemented an innovation contributing to MLCC.* The original sentence implies that the innovation directly advanced MLCC, but the results do not describe the extent of continuity. Did women meet a known midwife? I suggest changing this to focus more on the process of increasing care provided by midwives (in primary care), or to clearly illustrate how this innovation improved continuity of care.

Line 142, remove the comma after “participate”.

Please provide more details of the data collection process, how the participants were recruited, through email or phone?, whether the interviews were recorded (you only write about transcripts), and who conducted them.

Line 154-155, please give some suggestions on the questions or add a copy of the interview guide for the reader to better understand the concepts you were discussing with the participants.

Results

Please relocate Table 2 and the short description of the table to the Methods section, e.g line 148, and that Figure 1 be moved to the current position of Table 2, before describing the results. In addition, it would be beneficial to have a short description of the results before the constructs and teams are described, e.g moving the first part of the paragraph describing the theme in line 300-301 and adding something about financial aspects*. ”Trust emerged as a central and cross-cutting theme overarching the four core constructs of NPT, influencing the implementation and normalization of MLCC (see Figure 1).”*

This reordering would enhance the logic structure of the manuscript and improve coherence of the narrative flow.

I suggest writing the NPT construct before each theme, e.g *Coherence- Making sense of MLCC,* to facilitate the reader’s understanding.

Discussion

Trust and financial feasibility are discussed as fundamental for improving MLCC. This is well described and discussed with valuable insights. However, the discussion would be improved if you incorporated other references to it, as you have done in the Implications for practice section. Please revise these two sections (Discussion and Implications for practice) and merge them into one, including references of others’ work.

Strengths and limitations

The authors do not discuss the limitations of not examining the full implementation of a MLCC model, as only task shifting and shifting care to community settings are explored. Please add this as a limitation.

Line 448, please add “of” before the word trust (mechanism of trust).

Conclusion

The conclusion is well-written and provides a clear and concise summary of the findings, as well as what is required to strengthen the implementation of MLCC. However, I am hesitant about the phrasing of the first sentence (line 531-532), as the study does not examine the implementation of a full MLCC model. I recommend revising this paragraph to align with my overall comment on the manuscript, ensuring that the scope of the intervention is accurately represented.
