# Supplementary material for: Understanding the implementation of continuity-enhancing innovations as steps towards midwife-led continuity of care: A qualitative study using Normalization Process Theory
Source: PLoS One. 2026 Apr 21;21(4):e0347791. doi: 10.1371/journal.pone.0347791 (PMC13098977; doi:10.1371/journal.pone.0347791)
Supplement: Attachment — Submitted filename: Response to Reviewers.docx [file pone.0347791.s008.docx]

Amsterdam University Medical Centre (AUMC)

Vrije Universiteit Amsterdam

Department of Midwifery Science

Amsterdam Public Health

Van der Boechorststraat 7, 1081 BT Amsterdam

E: [r.simmelink@amsterdamumc.nl](mailto:r.simmelink@amsterdamumc.nl)

December 2025

Dear Editor, Dear Dr. Emily Chenette,

We would like to thank you and the reviewers for the valuable comments on our manuscript *‘Understanding the implementation of midwife-led continuity of care using the Normalization Process Theory’*, which we submitted for publication in PLOS One.

We have addressed the comments of the reviewers below. We have copied the comments of the reviewers and written our response *in italics*. Line numbers in our response refer to the marked copy of our manuscript. We hope you are in agreement with our edits to the original manuscript and look forward to hearing from you.

Kind regards, on behalf of all authors,

Renate Simmelink, MSc.

| **Reviewer 1** | |
| --- | --- |
| Thank you for the opportunity to review this paper using the Normalization Process Theory to understand the implementation of midwife-led continuity of care. The paper is well written, and using the NPT theory as a conceptual framework to understand how MLCC can become embedded and sustained in everyday work is a good approach. The results are significant for further implementation of MLCC. However, the authors need to clearly define that what you have assessed is not a full implementation of MLCC. While this nuance is mentioned in places- such as in the Supplementary material 1, where you note that ”some degree of MLCC had been implemented” – the introduction and methods sections currently give the impression that a full MLCC model was implemented. | *Thank you for your time and effort in reviewing our manuscript and providing valuable comments and suggestions, which helped us to improve the manuscript.*  *We agree that the original version of the manuscript could give the impression that full MLCC models had been implemented in the included networks. As suggested by the reviewer, we have now clarified throughout the manuscript that the study concerns innovations aimed at increasing continuity of care, rather than full implementation of MLCC. In addition, we changed the title and have added a sentence to the introduction to state that an immediate implementation of a complex intervention like MLCC is not feasible.*  *Please see page 2 lines 31 and 49, page 3 lines 72-74, page 4 line 92, page 6 line 148, page 7 line 157, page 22 lines 488 and 497, and page 25 line 568.* |
| The Introduction section gives an overview of the midwifery continuity of care (MLCC) model, but when reading the results I understand that this is more about task shifting or shifting care to community settings, and not really about implementing a MLCC model with a small group of midwives providing continuity 24/7 throughout the continuum of care with on-call for their women. The results are important and well-written but the Introduction needs to align better with the rest of the paper i.e. to focus more on midwifery led care and less on the results of the MLCC model (ref 2-4), as this is not the intervention you have explored. A MLCC model also have a strong midwifery philosophy with a focus on women-centered care and less use of unnecessary interventions, and I think the results do not align with the full concept of the model. I feel that using the word MLCC is not really correct when you only talk about part of this concept, i.e. shift in care towards midwife-led settings, and I wonder what the participants discuss when you describe that they had a strong conceptual support for MLCC. | *We agree that the innovations implemented in the included networks do not constitute a full MLCC model. We have carefully considered your suggestion to avoid the term MLCC, however, we respectfully believe that retaining MLCC as the overarching concept remains appropriate. We have seen that MLCC is a complex intervention that can only be implemented through incremental system changes. A new model needs to evolve through stepwise innovations, including task shifting and expanded midwife-led care. Therefore, the innovations studied here represent steps along the pathway towards MLCC. As stated above, we have clarified throughout the manuscript that our study focuses on continuity-enhancing innovations moving towards MLCC, rather than the implementation of a complete MLCC model. We hope the revisions make the alignment between the Introduction and Results clearer while allowing us to retain MLCC as the most appropriate overarching term and aim.* |
| In line 96-98, the authors describe how care is provided for low-risk women but it needs to be added the degree to which continuity is provided. Does the woman meet the same or a few midwives in this model? | *We agree that the original description of care for low-risk women did not sufficiently clarify the degree of continuity provided within the Dutch maternity care model. To address this, we have added an explanation in a textbox describing the Dutch maternity care system. In this textbox, we clarify that most community midwives work in group practices of varying size, with a supporting reference, see page 5 line 119. We believe this addition provides more clarity.* |
| In line 133-134, the authors need to clearly define what you mean with ”full continuity of care”. | *We agree that the phrase “full continuity of care” needs a clearer definition and may elicit questions. In the revised manuscript, we have adjusted this sentence to avoid implying the existence of a fully implemented MLCC model. Instead, we now specify that some regions had implemented broader organizational changes that increased continuity to a higher degree than the other specific innovations, see page 7 lines 160-162. The present description clarifies that these networks did not achieve full MLCC, but rather took more extensive steps toward improving continuity.* |
| It is not clear how healthcare is financed in the Netherlands, which makes it a bit confusing to read about financial aspects in the results. There is a short description of the financial system in the discussion (In systems based on fee-for-service payment models, like in the Netherlands,…) but this need to be described in the Introduction for the reader to understand the problems described in the Results. | *We agree that the original manuscript did not provide sufficient background on the organization and financing of Dutch maternity care to fully contextualize the findings noted in the Results. In response, we have added a textbox in the Introduction, see pages 5 and 6 from line 114, that explains:*   - *how maternity care is financed in the Netherlands for community midwives and the hospitals (lines 125-132);* |
| It is also not clear what role health insurance companies have. Please describe this in the background/context. | - *the role of health insurance companies (lines 126-127);* |
| In line 95, the authors also mentioned what professions are working in the Dutch maternity care system, but it would be helpful if you could describe what role the obstetric nurse has in relation to the midwife. | - *the role of the obstetric nurse in relation to the midwives (lines 123-124).* |
| Research question: Since the authors have only used one research question, I recommend changing it to an aim instead. | *We have revised the research question into an explicit aim in the Introduction, see page 5 lines 105-110.* |
| **Methods** | |
| Add a reference to the SRQR checklist. | *We have added the supporting reference, see page 6 line 144.* |
| Setting, participants and data collection: Line 123, …that implemented an innovation contributing to MLCC. The original sentence implies that the innovation directly advanced MLCC, but the results do not describe the extent of continuity. Did women meet a known midwife? I suggest changing this to focus more on the process of increasing care provided by midwives (in primary care), or to clearly illustrate how this innovation improved continuity of care. | *We agree that the sentence in the original manuscript may imply implementation of the full MLCC model in these maternity care networks. Therefore, we clarified that these networks implemented an MLCC-enhancing innovation, see page 6 line 148. In addition, we explained the aim of those MLCC-enhancing innovations in relation to the continuity of care context; providing an opportunity for women to continue receiving care from their own known midwifery team instead of a complete transfer to secondary care, see page 6 lines 149-151.* |
| Line 142, remove the comma after “participate”. | *We have removed the comma.* |
| Please provide more details of the data collection process, how the participants were recruited, through email or phone?, whether the interviews were recorded (you only write about transcripts), and who conducted them. | *We agree that this addition is essential to describe in the data collection process. Therefore, we have added how participants were invited (by email, telephone or in person). The interviews were conducted and recorded via Microsoft Teams and all conducted by the first two authors, see page 8 line 181-182.* |
| Line 154-155, please give some suggestions on the questions or add a copy of the interview guide for the reader to better understand the concepts you were discussing with the participants. | *We have added a translated interview guide as a supplemental material to the manuscript. We refer to this supplement on page 8 line 183.* |
| **Results** | |
| Please relocate Table 2 and the short description of the table to the Methods section, e.g line 148, and that Figure 1 be moved to the current position of Table 2, before describing the results. In addition, it would be beneficial to have a short description of the results before the constructs and teams are described, e.g moving the first part of the paragraph describing the theme in line 300-301 and adding something about financial aspects. ”Trust emerged as a central and cross-cutting theme overarching the four core constructs of NPT, influencing the implementation and normalization of MLCC (see Figure 1).” This reordering would enhance the logic structure of the manuscript and improve coherence of the narrative flow. | *The sequence of the Results has frequently been revised and discussed among the authors. We believe that starting with the NPT constructs is in line with the aim of the study and builds towards the overarching themes. However, we agree that it can be helpful to address these overarching themes shortly in the beginning of the Results to enhance the logic structure of the manuscript and to provide the reader with some narrative flow. Therefore, we relocated lines 334-335 from the original manuscript to before the NPT constructs, including the financial aspect, together with Figure 1, see page 11 lines 222-223.* |
| I suggest writing the NPT construct before each theme, e.g Coherence- Making sense of MLCC, to facilitate the reader’s understanding. | *We have added the NPT constructs before each theme, see page 11 line 227, page 12 line 248, page 13 line 277, and page 15 line 306.* |
| **Discussion** | |
| Trust and financial feasibility are discussed as fundamental for improving MLCC. This is well described and discussed with valuable insights. However, the discussion would be improved if you incorporated other references to it, as you have done in the Implications for practice section. Please revise these two sections (Discussion and Implications for practice) and merge them into one, including references of others’ work. | *In our manuscript, the “Implications for practice” is intentionally presented as a sub-section within the broader Discussion, rather than as a separate section. We have added the subheadings solely for readability. For that reason, we have kept these parts distinct in structure but connected in content.* |
| **Strengths and limitations** | |
| The authors do not discuss the limitations of not examining the full implementation of a MLCC model, as only task shifting and shifting care to community settings are explored. Please add this as a limitation. | *We have added the limitation of not being able to examine the implementation of a full MLCC model because this appears to be difficult in practice, see page 23 lines 509-510.* |
| Line 448, please add “of” before the word trust (mechanism of trust). | *We have added “of” before trust, see page 22 line 483.* |
| **Conclusion** | |
| The conclusion is well-written and provides a clear and concise summary of the findings, as well as what is required to strengthen the implementation of MLCC. However, I am hesitant about the phrasing of the first sentence (line 531-532), as the study does not examine the implementation of a full MLCC model. I recommend revising this paragraph to align with my overall comment on the manuscript, ensuring that the scope of the intervention is accurately represented. | *As stated in our previous response, we have now clarified throughout the manuscript that our study focusses on MLCC-enhancing initiatives, rather than full implementation of MLCC. This has also been clarified in the conclusions, see page 25 line 568.* |

| **Reviewer 2** | |
| --- | --- |
| Thank you for the opportunity to review this interesting and important study addressing the implementation of MLCC within existing care systems. This work contributes to the important body of knowledge on how evidence-based care can be provided to women. The manuscript is well written and employs Normalization Process Theory to explore the implementation process. The inclusion of multiple stakeholders offers a broad range of perspectives, which enriches the understanding of MLCC implementation. | *Thank you for seeing the value of our work and helping us to improve our manuscript with your valuable comments and suggestions.* |
| A more detailed description of how the Framework Method was used in the analysis would have been beneficial. As a reader, I find it difficult to follow what the authors have done and how they have done it. Consequently, I consulted the cited reference for the method (14) to gain a basic understanding, as I am not familiar with it. In that paper, the method is described in six steps, and the analysis is conducted using a matrix. Did you use a matrix? Is it included anywhere in the submission? Would it make sense to publish it, or provide examples of how you worked with this matrix? Alternatively, is the NPT your matrix? Including such material might help readers follow the description and understand the details of the analysis. | *We have used the Framework Method as an overarching analytic structure, with the four core constructs of the NPT functioning as our analytical framework. Within this structure, we remained open to identifying additional themes that emerged from the data. In our study, NPT served as the organizing framework rather than a matrix in the traditional sense of the Framework Method, so we did not construct a full matrix as described in Gale et al. (2013). Our coding process did involve systematically indexing data to these constructs, as well as to inductively derived themes. We believe this is now more clear with the additions we have made on page 8 lines 189-190.* |
| **Methods** | |
| I find it somewhat difficult to follow the description of the setting and to determine whether all stakeholders are involved in, or working closely with, a setting that provides MLCC. In the Methods section, under Setting, participants and data collection (line 123), the phrase “innovation contributing to MLCC”, and later (line 134) “networks that appeared to come close to achieving full continuity of care” and “enhanced continuity by shifting care from obstetrician-led to midwife-led models” seem rather vague. A model can be midwife-led and continuity can be increased without meeting the criteria for classification as an MLCC model (as outlined in your Introduction, line 55). Do you mean a modified MLCC model? Do these innovations indicate that MLCC was achieved, or were they part of an implementation process aimed at establishing an MLCC model? | *We agree that the original version of the manuscript could give the impression that full MLCC models had been implemented in the included networks. Based on your comment, we have clarified throughout the manuscript that the study concerns innovations aimed at increasing continuity of care, rather than full implementation of MLCC. In addition, we changed the title and have added a sentence to the introduction to state that an immediate implementation of a complex intervention like MLCC is not feasible. The present description clarifies that the included networks did not achieve full MLCC, but rather took steps toward improving continuity.*  *Please see page 2 lines 31 and 49, page 3 lines 72-74, page 4 line 92, page 6 line 148, page 7 line 157, page 22 lines 488 and 497, and page 25 line 568.*  *Moreover, we have reformulated the sentence about “networks that appeared to come close to achieving full continuity of care”, see page 7 lines 160-162. Those networks made broader organizational changes to achieve a higher degree of continuity.* |
| **Results** | |
| In the Results section, under Financial impact of MLCC (line 358), the statement “The implementation of MLCC, along with the associated potential shift in care, carries significant financial implications” raises some similar questions. If MLCC is implemented, does this not necessarily entail a shift in care? Why is this described as a ‘potential’ shift? | *We agree that the term ‘potential’ is misplaced in this context. It has been removed, see page 18 line 393.* |
| Figure 1. is presented in the middle of the Results section. To provide an overview of your findings more effectively, it might be useful to present the figure earlier or to describe your findings in terms of themes, their names and interrelations. The current structure of the Results section introduces the reader directly to one of the four core concepts of the NPT. However, in Figure 1, these core concepts do not appear to interrelate as described in the cited framework. | *We agree that the sequence of the Results is challenging. It has frequently been revised and discussed among the authors. To enhance the* *logic structure of the manuscript we have added a short description of the Results before the NPT constructs are described, see page 11 lines 222-223. Furthermore, we relocated Figure 1 to the beginning of the Results section.* |
| It might have been helpful to include an introduction explaining how the authors conceptualised the changed relationships of the core concepts and the introduction of the additional findings of financial system and organizational system surrounding the NPT core concepts and the central concept of ‘trust’. This is for example described with the following phrase in the Discussion, (line 149) ‘Our findings demonstrate how trust and financial feasibility can be seen as cross-cutting and foundational mechanisms …’ | *In order to conceptualize the relationships of the core constructs and the central overarching themes trust and financial feasibility we have moved Figure 1 to the beginning of the Results section.* |
| **Discussion** | |
| Additionally, could you elaborate on what you mean by the concept of “trust” referred to in this context? Is it trust between stakeholders and their collaboration, trust in the concept of MLCC, trust in the shift of care systems, or something else? | *We have added that ‘trust’ refers to as ‘trust between stakeholders’ in this context, see page 20 line 453, and page 25 line 570.* |
