# Supplementary material for: Understanding the implementation of continuity-enhancing innovations as steps towards midwife-led continuity of care: A qualitative study using Normalization Process Theory
Source: PLoS One. 2026 Apr 21;21(4):e0347791. doi: 10.1371/journal.pone.0347791 (PMC13098977; doi:10.1371/journal.pone.0347791)
Supplement: Attachment — Submitted filename: 260111_Reviewers comments_SdW.docx [file pone.0347791.s007.docx]

**11.01.2026**

**Reviewer’s Comments on the Manuscript: “Understanding the implementation of midwife-led continuity of care using the Normalization Process Theory”**

Thank you for the opportunity to review this interesting and relevant paper. The manuscript addresses an important gap in the literature. Below are my detailed comments.

**Abstract**

The abstract should be revised after incorporating changes to the main manuscript based on reviewer recommendations.

**Background**

The background section would benefit from clearer definitions and explanations. Currently, the understanding of MLCC, its strengths, and barriers to implementation is left largely to the reader. Please clarify:

- What exactly are *Maternity Care Networks*?
- What is the MLCC model within these networks?
- How is MLCC embedded in the referral system?
- Who is involved at the point of referral?
- What are the expected outcomes of MLCC?

**Methods**

**Setting, Participants, and Data Collection**
Information about Maternity Care Networks is difficult for readers unfamiliar with the context. Consider moving this information to the background section.

Additionally:

- Explain who was recruited and how recruitment occurred within these networks.
- Provide a sampling matrix to illustrate the purposive sampling strategy. This will help readers understand purposive sampling strategy and will be useful when presenting results.

**Data Analysis**
The description of the Thematic Framework approach does not align with corresponding methods. Please include the following steps if this approach was used:
(1) Transcription; (2) Familiarisation; (3) Coding; (4) Developing an analytic framework; (5) Indexing; (6) Charting; and (7) Interpreting the data.

**Results**

**Participant Characteristics**
Clarify participant characteristics within the context of Maternity Care Networks. This links back to the need for a sampling matrix and details on theoretical sampling.

**Overview of Results**
The emerging framework within NPT is not presented. An illustration at the beginning of the results section showing the themes and their interpretation in the MLCC context would greatly improve clarity. Currently, the relationship between themes and the NPT framework is difficult to follow.

**Discussion**

The discussion lacks structure and does not align with SRQR guidelines for integrating results with existing evidence. This section requires substantial revision to ensure clarity and coherence.
