# Supplementary material for: Understanding the implementation of continuity-enhancing innovations as steps towards midwife-led continuity of care: A qualitative study using Normalization Process Theory
Source: PLoS One. 2026 Apr 21;21(4):e0347791. doi: 10.1371/journal.pone.0347791 (PMC13098977; doi:10.1371/journal.pone.0347791)
Supplement: Attachment — Submitted filename: Response_to_Reviewers_auresp_2.docx [file pone.0347791.s010.docx]

Amsterdam University Medical Centre (AUMC)

Vrije Universiteit Amsterdam

Department of Midwifery Science

Amsterdam Public Health

Van der Boechorststraat 7, 1081 BT Amsterdam

E: [r.simmelink@amsterdamumc.nl](mailto:r.simmelink@amsterdamumc.nl)

February 2025

Dear Editor, dear Dr. Emily Chenette,

We would like to thank you and the reviewers for the valuable comments on our manuscript *‘Understanding the implementation of continuity-enhancing innovations as steps towards midwife-led continuity of care: a qualitative study using Normalization Process Theory’*, which we submitted for publication in PLOS One.

We have addressed the comments of the reviewers below. We have copied the comments of the reviewers and written our response *in italics*. Line numbers in our response refer to the marked copy of our manuscript. We hope you are in agreement with our edits to the original manuscript and look forward to hearing from you.

Kind regards, on behalf of all authors,

Renate Simmelink, MSc.

| **Reviewer 2** | |
| --- | --- |
| **Discussion** | |
| Thank you for the opportunity to read the revised version of your manuscript. It is evident that you have invested considerable effort in addressing the previous comments, and several sections have been strengthened as a result. I do, however, have one remaining concern that I do not feel has been fully resolved: the positioning of your findings in relation to the existing body of research.  While the discussion section now includes reflections on clinical implications, as well as strengths and limitations, the subsection that situates your results within the context of prior studies remains rather limited. If this brevity reflects a genuine lack of previous research in the area, I encourage you to state this explicitly. Otherwise, expanding this part of the discussion would help readers better understand how your findings align with, diverge from, or extend what is already known. | *We understand the potential confusion in the initial version of the discussion, where several statements summarizing our results were presented in a general manner without indicating that they stemmed from our own findings. We have now revised this section to explicitly attribute these points to our study results, see lines 463-490. Furthermore, we have expanded the ‘Implications for practice’ section by integrating additional relevant literature, and situating our findings more clearly in relation to prior research and highlighting how our study extends existing knowledge, see lines 529-598.* |
| Regarding line 465, I would like to draw your attention to what appears to be a typographical error. Did you intend to write “country” rather than “county”? Clarifying this would help avoid potential misunderstanding. | *Thank you for your attentiveness, it should be indeed “country”.* |
| In addition, I am still unable to locate the completed SRQR checklist. If it has been uploaded, it may be helpful to indicate its exact location in the submission system. If it has not yet been provided, please ensure that the filled checklist is included with the revised materials. | *The SRQR checklist had indeed not been included in the original submission, but has now been added.* |
| **Reviewer 3** | |
| **Abstract** |  |
| The abstract should be revised after incorporating changes to the main manuscript based on reviewer recommendations. | *After the revisions, we checked and updated the abstract to ensure that it still accurately reflects the content of the revised manuscript.* |
| **Background** |  |
| The background section would benefit from clearer definitions and explanations. Currently, the understanding of MLCC, its strengths, and barriers to implementation is left largely to the reader. Please clarify: • What exactly are Maternity Care Networks? • What is the MLCC model within these networks? • How is MLCC embedded in the referral system? • Who is involved at the point of referral? • What are the expected outcomes of MLCC? | *We acknowledge the challenge of explaining the Dutch system clearly in an international context. We have added a textbox within the introduction for further clarification. In doing so, we outline a more comprehensive picture of the organization of maternity care in the Netherlands. To give a better understanding of the scope and structure of a maternity care network, we have included additional explanations, see lines 121-126:*  *“Maternity care networks are a formal regional partnership between a hospital and the surrounding community-based midwifery practices. Within a maternity care network, hospital-based care providers and community midwives work closely together to coordinate maternity care, ensuring clear referral pathways, and shared responsibility throughout pregnancy, childbirth, and the postnatal period. Community-based midwifery practices may be affiliated with more than one maternity care network.”*  *Women who develop an intermediate or high risk for complications are referred to obstetrician-led hospital care. This referral is initiated by community midwives and has implications for the continuity of care (see lines 101-105):*  *“Women who develop an intermediate or high risk for complications are referred to obstetrician-led hospital care, often resulting in a change of care professionals. Such referral systems can disrupt continuity of care, as women often need to transition between different care settings and professionals throughout their pregnancy, during childbirth, or in the postpartum period.”*  *We elaborate on the expected outcomes of MLCC in lines 64-69.* |
| **Methods** | |
| **Setting, Participants, and Data Collection** Information about Maternity Care Networks is difficult for readers unfamiliar with the context. Consider moving this information to the background section.  Additionally:   - Explain who was recruited and how recruitment occurred within these networks. - Provide a sampling matrix to illustrate the purposive sampling strategy. This will help readers understand purposive sampling strategy and will be useful when presenting results. | *To clarify the structure of maternity care networks we added additional background information within the textbox “organization of maternity care in the Netherlands” (see lines 121-126).*  *In response to your first comment, we have expanded the Methods section to more clearly explain who was recruited and when we stopped sampling. Recruitment is explained in lines 178-188:*  *“Participants were recruited through an initial purposive sampling strategy. The first participants invited were members of regional MLCC implementation project groups, as they were directly involved in the design and implementation of the innovation. As data collection progressed, theoretical sampling was applied, meaning that additional participants were selectively invited based on emerging findings from earlier interviews. By iteratively refining participant selection, key perspectives and experiences relevant to the implementation of MLCC were deeper explored. Sampling continued until sufficient information had been obtained within each region to capture experiences relevant to the constructs of NPT, and no substantively new insights emerged in subsequent interviews. Participants were invited by email, telephone or in person. Semi-structured interviews were conducted and recorded via Microsoft Teams by RS and AP, using a topic list based on sensitizing concepts considered relevant in literature (supplement material 2).”*  *We have revised the tables in the Results section to provide a clear overview of all participants per region. These tables are intended to illustrate the sampling strategy and to support transparency regarding the composition of the sample across regions. We believe this provides readers with a clear overview of the sampling approach and facilitates interpretation of the findings. See table 1 and table 2 on page 11.* |
| **Data Analysis** The description of the Thematic Framework approach does not align with corresponding methods. Please include the following steps if this approach was used: (1) Transcription; (2) Familiarisation; (3) Coding; (4) Developing an analytic framework; (5) Indexing; (6) Charting; and (7) Interpreting the data. | *We agree that greater clarity regarding the analytical steps of the Thematic framework is warranted. All stages of the Framework method were undertaken. However, in our study, we applied the Framework method in a theory-informed manner, using the four core constructs of NPT as an a priori analytical framework. This reflects a predominantly deductive approach, while remaining open to inductively generated themes. We have now revised this part of the Method section to clearly describe the analytical steps and to further clarify how the NPT framework guided analysis, see lines 194-210.* |
| **Results** | |
| **Participant Characteristics** Clarify participant characteristics within the context of Maternity Care Networks. This links back to the need for a sampling matrix and details on theoretical sampling. | *As stated above, we have revised the tables in the Results section to provide a clear overview of participant characteristics per region. Moreover, we expanded the section describing the sampling method for greater clarity.* |
| **Overview of Results** The emerging framework within NPT is not presented. An illustration at the beginning of the results section showing the themes and their interpretation in the MLCC context would greatly improve clarity. Currently, the relationship between themes and the NPT framework is difficult to follow. | *The themes presented in the results correspond to the core constructs of NPT. During the analysis, it became apparent that two themes (financial feasibility, and trust) emerged within each construct. For this reason, we elected to describe them separately. To clarify more that the themes correspond to the core construct of NPT we added figure 1 at the beginning of the Results section and named the NPT construct at the beginning of each paragraph (coherence, cognitive participation, collective action, reflexive monitoring). We inserted a clarifying remark to further elucidate this, see line 238-239:*  *“Trust and financial feasibility emerged as central and cross-cutting themes overarching the four core constructs of NPT, influencing the implementation and normalization of MLCC (see Figure 1).”* |
| **Discussion** | |
| The discussion lacks structure and does not align with SRQR guidelines for integrating results with existing evidence. This section requires substantial revision to ensure clarity and coherence. | *We have separated our Discussion section into a summary of our main findings, strengths and limitations, and implications for practice. We understand the potential confusion in the initial version of the discussion, where several statements summarizing our results were presented in a general manner without indicating that they stemmed from our own findings. We have now revised this section to explicitly attribute these points to our study results, see lines 463-490. In the implications for practice section, we explicitly integrated our results with relevant prior literature, highlighting where our findings align with, extend, or differ from existing evidence, see lines 529-598. We believe that the discussion section now aligns with the SRQR guidelines.* |
