# Supplementary material for: Understanding the implementation of continuity-enhancing innovations as steps towards midwife-led continuity of care: A qualitative study using Normalization Process Theory
Source: PLoS One. 2026 Apr 21;21(4):e0347791. doi: 10.1371/journal.pone.0347791 (PMC13098977; doi:10.1371/journal.pone.0347791)
Supplement: Attachment — Submitted filename: Peer review 3 March 2026.docx [file pone.0347791.s009.docx]

**Peer-review of ’Understanding the implementation of continuity-enhancing innovations as steps towards midwife-led continuity of care: a qualitative study using Normalization Process Theory’**

**Minor issues to address**

**Abstract**

Methods

To improve language, remove ’the comma’ and ’were’ in the following paragraph: *Stakeholders invited to participate in semi-structured interviews****, were***

*community midwives, hospital-based midwives, obstetricians, managers, an obstetric*

*nurse, and healthcare insurance company employees.*

New suggestion: *Stakeholders invited to participate in semi-structured interviews* ***included*** *community midwives, hospital-based midwives, obstetricians, managers, an obstetric nurse, and healthcare insurance company employees.*

**Discussion**

Line 456- add the word ’elements’ in this paragraph to point out that it is not a full MLCC model that is tested. This will also be in line with the aim of the study.

New suggestion: *In this study, the NPT framework was used to examine how MLCC* ***elements were*** *implemented and sustained….*

The layout of the discussion could still be improved. According to the SRQR checklist, the discussion should include i) *integration with prior work, implications, transferability, and contribution(s) to the field, and* ii) *limitations.*

I have previously suggested merging the Discussion and Implications for practice sections, as the first part includes no references, which is a bit odd. However, if you prefer to keep them separate, I strongly encourage you to move the Strengths and Limitations section to the end, after the Implications for practice section, so that the discussion remains grouped with the text that includes other references.
