# Supplementary material for: Understanding the implementation of continuity-enhancing innovations as steps towards midwife-led continuity of care: A qualitative study using Normalization Process Theory
Source: PLoS One. 2026 Apr 21;21(4):e0347791. doi: 10.1371/journal.pone.0347791 (PMC13098977; doi:10.1371/journal.pone.0347791)
Supplement: Attachment — Submitted filename: Response_to_Reviewers_auresp_3.docx [file pone.0347791.s011.docx]

Amsterdam University Medical Centre (AUMC)

Vrije Universiteit Amsterdam

Department of Midwifery Science

Amsterdam Public Health

Van der Boechorststraat 7, 1081 BT Amsterdam

E: [r.simmelink@amsterdamumc.nl](mailto:r.simmelink@amsterdamumc.nl)

March 2026

Dear Editor, dear Dr. Emily Chenette,

We would like to thank you and the reviewers for the valuable comments on our manuscript *‘Understanding the implementation of continuity-enhancing innovations as steps towards midwife-led continuity of care: a qualitative study using Normalization Process Theory’*, which we submitted for publication in PLOS One.

We have addressed the comments of the reviewers below. We have copied the comments of the reviewers and written our response *in italics*. Line numbers in our response refer to the marked copy of our manuscript. We hope you are in agreement with our edits to the manuscript and look forward to hearing from you.

Kind regards, on behalf of all authors,

Renate Simmelink, MSc.

| **Reviewer 1** | |
| --- | --- |
| **Abstract** | |
| Methods  To improve language, remove ’the comma’ and ’were’ in the following paragraph:  Stakeholders invited to participate in semi-structured interviews**, were** community midwives, hospital-based midwives, obstetricians, managers, an obstetric nurse, and healthcare insurance company employees.  New suggestion: Stakeholders invited to participate in semi-structured interviews **included** community midwives, hospital-based midwives, obstetricians, managers, an obstetric nurse, and healthcare insurance company employees. | *We have revised the sentence accordingly and removed the comma and ‘were’ to improve the language, see page 2 line 37.* |
| **Discussion** | |
| Line 456- add the word ’elements’ in this paragraph to point out that it is not a full MLCC model that is tested. This will also be in line with the aim of the study.  New suggestion: In this study, the NPT framework was used to examine how MLCC **elements were** implemented and sustained…. | *We have added the word ‘elements’ to make clear that not a full MLCC model was implemented, see page 20 line 455.* |
| The layout of the discussion could still be improved. According to the SRQR checklist, the discussion should include i) *integration with prior work, implications, transferability, and contribution(s) to the field, and* ii) *limitations.*  I have previously suggested merging the Discussion and Implications for practice sections, as the first part includes no references, which is a bit odd. However, if you prefer to keep them separate, I strongly encourage you to move the Strengths and Limitations section to the end, after the Implications for practice section, so that the discussion remains grouped with the text that includes other references. | *We have revised the structure of the Discussion section. The subsection ‘Strengths and Limitations’ has been moved to the end of the Discussion, following the ‘Implications for Practice’ subsection, so that the summary of results and integration of our findings with existing literature remain grouped together.*  *We have retained a brief paragraph summarizing the main findings before integrating them with the existing literature. This structure is commonly used in qualitative research articles to orient the reader to the key findings before engaging in interpretation and comparison with prior work. Similar Discussion structures can be found in other articles published in this journal, where the Discussion likewise begins with a short summary of the findings before engaging with the literature. See for example:* [*The use of international comparison as interactive teaching method in pharmacy education \| PLOS One*](https://journals.plos.org/plosone/article?id=10.1371/journal.pone.0338269)*. However, the opening paragraph has been shortened by removing several sentences, in order to improve readability, see pages 20&21 lines 455-480.* |
| **Reviewer 2** | |
| I still cannot locate the filled SRQR checklist. Although the authors state that it has been uploaded, it does not appear in the PDF provided for review. It is possible that this may be related to the submission system. | *The completed SRQR checklist was uploaded during submission. It is therefore possible that this issue is related to the submission system. To ensure accessibility for the reviewers, we have uploaded the checklist again with the revised submission.* |
| I also believe that the manuscript would benefit from being shorter and more concise; however, if there are no word limits, this may ultimately be a matter of preference. After reading the full Results section, I find it difficult to identify the main findings of the study. While the conclusion and abstract are clear on this point, I am uncertain whether all the information presented across nine pages of results is necessary for the readers. | *In response to this suggestion, we have shortened the Results section by removing several explanatory sentences while preserving the core findings.* |
| **Discussion** | |
| In the Discussion section, lines 456–481 appear to continue the presentation of results rather than offering interpretation. To strengthen the manuscript, you may wish to revise this section to connect it with the contextualisation of your findings in relation to existing literature. | *In line with this comment, we have shortened the opening paragraph of the Discussion section by removing several sentences. We choose to retain a brief paragraph summarizing the main findings, which is more commonly used in qualitative research. Similar Discussion structures can be found in other articles published in this journal, where the Discussion likewise begins with a short summary of the findings before engaging with the literature. We have, however, strengthened the connection with existing literature in the subsequent paragraphs, by relocating the ‘Strengths and Limitations’ section to the end of the Discussion.* |
| Moreover, I am unfamiliar with a strengths‑and‑limitations section in a qualitative study that does not address aspects of trustworthiness. Consider elaborating on credibility, dependability, confirmability, or transferability to enhance the rigor of the manuscript. | *We have expanded the Strengths and Limitations section to more explicitly address aspects of trustworthiness in qualitative research, including credibility, confirmability, and transferability. On page 26 lines 604-607 we added:*  *‘Credibility was further strengthened through the inclusion of a wide range of stakeholders across different professional groups and maternity care networks, enabling triangulation of perspectives. In addition, the multidisciplinary composition of the research team facilitated critical reflection, thereby enhancing confirmability of the findings.’*  *Furthermore, we made additions in lines 610 and 621.* |
